# Abdominopelvic trauma: from anatomical to anatomo-physiological classification

**DOI:** 10.1186/s13017-018-0211-4

**Published:** 2018-10-31

**Authors:** Federico Coccolini, Fausto Catena, Yoram Kluger, Massimo Sartelli, Gianluca Baiocchi, Luca Ansaloni, Ernest Eugene Moore

**Affiliations:** 1 General, Emergency and Trauma Surgery Department, Bufalini Hospital, Viale Ghirotti 268, 47521 Cesena, Italy; 2 Emergency Surgery Department, Parma Maggiore Hospital, Parma, Italy; 3 Division of General Surgery, Rambam Health Care Campus Haifa, Haifa, Israel; 4 General Surgery Department, Macerata Hospital, Macerata, Italy; 5 Department of Clinical and Experimental Sciences, University of Brescia, Brescia, Italy; 6 Trauma Surgery, Denver Health, Denver, CO USA

**Keywords:** Trauma, Classification, Physiology, Stratification, Decision, Flow-chart, Algorithm, Guidelines, Spleen, Liver, Pelvis, Kidney, Polytrauma, Hybrid, Endovascular, Bleeding, Management, Emergency general surgery

## Abstract

Abdominopelvic trauma has been for decades classified with the AAST-OIS (American Association for the Surgery of Trauma—Organ Injury Scale) classification. It has represented a milestone. At present, the medical evolutions in trauma management allowed an incredible progress in trauma decision-making and treatment. Non-operative trauma management has been widely applied. The interventional radiological procedures and the modern conception of Hybrid and Endovascular Trauma and Bleeding Management (EVTM) led to good results in increasing the rate of patients managed non-operatively, opening new scenarios and options. Even severe anatomical lesions in hemodynamically stable patients can be safely managed non-operatively. The driving issue in deciding for the best treatment is anatomy, as well as physiology, for the patient physiological derangement grade is even more important. The emergency general surgeon must be prepared in those pathophysiological issues that play the pivotal role in the team management of trauma patients. Moreover, the classification of trauma patients cannot only remain anchored to anatomical lesions. The necessity to follow the modern possibilities of treatment imposes addressing trauma using a classification based on anatomical lesions and on the physiological status of the patient.

## Dear Editor,

We would like to present here the new classifications elaborated by the WSES expert panel regarding the abdominopelvic trauma. Abdominopelvic trauma has been for decades classified with the AAST-OIS classification that has represented a milestone [1]. Since its publication, it has been the only existing classification and it demonstrated to be effective and useful [2, 3]. The primary aim of AAST-OIS classification was to allow comparisons within different cohorts of patients. However, it was used to drive management algorithms that show some criticisms. Timely in 1994, when this anatomical classification was published, even CT-scan started to be diffusely used to diagnose and stratify trauma lesions and patients. This temporal synergism increased the efficiency of both tools in graduating preoperatively trauma injuries and comparing different cohorts. Actually, at that time, anatomy was one of the most reliable factors in classifying and deciding the treatment of trauma victims.

At present, the medical evolutions in trauma management allowed an incredible progress in trauma decision-making and treatment. Non-operative trauma management (NOM) has been widely applied with improved outcome, avoiding the operative intervention in several patients who previously would have undergone surgery. In fact, the interventional radiological procedures and the modern conception of Hybrid and Endovascular Trauma and Bleeding Management (EVTM) led to good results in increasing the rate of patients managed non-operatively, opening new scenarios and options in trauma patients management.

Since, at present, even severe anatomical lesions in hemodynamically stable patients can be safely managed non-operatively, the driving issue in deciding for the best treatment is anatomy, as well as physiology, for the patient physiological derangement grade is even more important.

As emergency general surgeons, we now are living in a new era of trauma management: we more often have to decide to be “non-operative”. In order to work this way, it is necessary to know the pathophysiology and the metabolic processes triggered by traumatic hits, on top of a wide anatomical knowledge. This evolution is pushing us to take into consideration a new approach to traumatized patients. The introduction of hybrid operating rooms will progressively delocate the treatment, at least of the more severe trauma patients, outside the proper and classical trauma surgeon view. The central role of the trauma surgeon has progressively changed not excluding him/her from the decision-making process, but actually involving him/her more and more deeply into highly complex decisions: the decision “not to operate”. To be “free” to decide in this new deal of trauma management, acute care surgeons must be, as said, even more prepared regarding pathophysiological issues, occupying the pivotal role in the team management of trauma patients.

Moreover, in all those countries or situations where the anatomical grade of lesions cannot be known preoperatively, due to the scarce possibility to access to CT-scan, physiology is the most important and often the only driving issue. In these cases, the anatomo-physiological classification would be more effective in driving management.

As a conclusion, the classification of trauma patients cannot remain anchored only to anatomical lesions. The necessity to follow the modern possibilities of treatment imposes addressing trauma using a classification based on anatomical lesions and on the physiological status of the patient [4–6].

In abdominopelvic trauma, the most involved, risky, and bleeding organs are the liver, the spleen, the kidneys, and the pelvic ring; for this reason, these have been the four topics of the new classification system.

The classifications were firstly discussed through the Delphi model within the expert panel. During the last three WSES world congresses, consensus conferences were held to approve the classifications together with the guidelines. The panel reached an agreement regarding the definition of hemodynamic instability as follows: “Hemodynamic instability is considered the condition in which the patient has an admission systolic blood pressure < 90 mmHg with evidence of skin vasoconstriction (cool, clammy, decreased capillary refill), altered level of consciousness and/or shortness of breath, or > 90 mmHg but requiring bolus infusions/transfusions and/or vasopressor drugs and/or admission base excess (BE) > − 5 mmol/l and/or shock index > 1 and/or transfusion requirement of at least 4–6 units of packed red blood cells within the first 24 h; moreover, transient responder patients (those showing an initial response to adequate fluid resuscitation and then signs of ongoing loss and perfusion deficits) and more in general those responding to therapy but not amenable of sufficient stabilization have undergone interventional radiology treatments” [5].

The four classifications for abdominopelvic lesions specifically about liver, spleen, kidney, and pelvic ring injuries resulted as follows.

## Liver injuries

The WSES classification divides hepatic injuries into three degrees considering the AAST-OIS classification and the hemodynamic status (Table 1) [4]:

- Minor (WSES class I)
- Moderate (WSES class II)
- Severe (WSES class III and IV)

**Table 1:** Liver trauma classification

	WSES grade	AAST	Haemodynamic
Minor	WSES grade I	I–II	Stable
Moderate	WSES grade II	III	Stable
Severe	WSES grade III	IV–V	Stable
	WSES grade IV	Any	Unstable

*WSES* World Society of Emergency Surgery, *AAST* American Association for the Surgery of Trauma

### Minor hepatic injuries

*WSES class I* includes hemodynamically stable AAST-OIS grade I–II blunt and penetrating lesions.

### Moderate hepatic injuries

*WSES class II* includes hemodynamically stable AAST-OIS grade III blunt and penetrating lesions.

### Severe hepatic injuries

*WSES class III* includes hemodynamically stable AAST-OIS grade IV–VI blunt and penetrating lesions.

*WSES class IV* includes hemodynamically unstable AAST-OIS grade I–VI blunt and penetrating lesions.

## Spleen injuries

The WSES classification divides spleen injuries into three degrees considering the AAST-OIS classification and the hemodynamic status (Table 2) [5]:

- Minor (WSES class I)
- Moderate (WSES class II and III)
- Severe (WSES class IV)

**Table 2:** Spleen trauma classification

	WSES class	AAST	Haemodynamic
Minor	WSES I	I–II	Stable
Moderate	WSES II	III	Stable
	WSES III	IV–V	Stable
Severe	WSES IV	I–V	Unstable

*WSES* World Society of Emergency Surgery, *AAST* American Association for the Surgery of Trauma

### Minor spleen injuries

*WSES class I* includes hemodynamically stable AAST-OIS grade I–II blunt and penetrating lesions.

### Moderate spleen injuries

*WSES class II* includes hemodynamically stable AAST-OIS grade III blunt and penetrating lesions.

*WSES class III* includes hemodynamically stable AAST-OIS grade IV–V blunt and penetrating lesions.

### Severe spleen injuries

*WSES class IV* includes hemodynamically unstable AAST-OIS grade I–V blunt and penetrating lesions.

## Kidney injuries

The WSES classification divides kidney injuries into three degrees considering the AAST-OIS classification, the hemodynamic status, and the eventual associated kidney vascular lesions (Table 3):

- Minor (WSES class I)
- Moderate (WSES class II)
- Severe (WSES class III and IV)

**Table 3:** Kidney trauma classification

	WSES grade	AAST	Haemodynamic
Minor	WSES grade I	I–II	Stable
Moderate	WSES grade II	III or segmental vascular injuries	Stable
Severe	WSES grade III	IV–V or any grade parenchymal lesion with main vessels dissection/occlusion	Stable
	WSES grade IV	Any	Unstable

*WSES* World Society of Emergency Surgery, *AAST* American Association for the Surgery of Trauma

### Minor kidney injuries

*WSES class I* includes hemodynamically stable AAST-OIS grade I–II blunt and penetrating lesions.

### Moderate kidney injuries

*WSES class II* includes hemodynamically stable AAST-OIS grade III blunt and penetrating lesions or segmental vascular injuries.

### Severe kidney injuries

*WSES class III* includes hemodynamically stable AAST-OIS grade IV–V blunt and penetrating lesions or any grade parenchymal lesion with main vessel dissection/occlusion.

*WSES class IV* includes hemodynamically unstable AAST-OIS grade I–V blunt and penetrating lesions.

## Pelvic ring injuries

The WSES classification divides pelvic ring injuries into three degrees considering the Young-Burgees classification, the hemodynamic status, and the mechanical status (Table 4) [6]:

- *Minor* (WSES class I) comprising hemodynamically and mechanically stable lesions
- *Moderate* (WSES class II, III) comprising hemodynamically stable and mechanically unstable lesions
- *Severe* (WSES class IV) comprising hemodynamically unstable lesions independently from mechanical status.

**Table 4:** Pelvic ring injuries classification

	WSES grade	Young-Burgees classification	Haemodynamic	Mechanic
Minor	WSES grade I	APC I–LC I	Stable	Stable
Moderate	WSES grade II	LC II/III–APC II/III	Stable	Unstable
	WSES grade III	VS	Stable	Unstable
Severe	WSES grade IV	Any	Unstable	Any

*APC* antero-posterior compression, *LC* lateral compression, *VS* vertical shear, *CM* combined mechanism

### Minor pelvic injuries

*WSES class I* includes APC I and LC I hemodynamically stable pelvic ring injuries.

### Moderate pelvic injuries

*WSES class II* includes APC II–III and LC II–III hemodynamically stable pelvic ring injuries.

*WSES class III* includes VS and CM hemodynamically stable pelvic ring injuries.

### Severe pelvic injuries

*WSES class IV* includes any hemodynamically unstable pelvic ring injuries.

## Abbreviations

AAST: American Association for the Surgery of Trauma
APC: Antero-posterior compression
CM: Combined mechanism
EVTM: Hybrid and Endovascular Trauma and Bleeding Management
LC: Lateral compression
NOM: Non-operative trauma management
OIS: Organ Injury Scale
VS: Vertical shear
WSES: World Society of Emergency Surgery

## References

[1] Moore EE, Shackford SR, Pachter HL, McAninch JW, Browner BD, Champion HR, Flint LM, Gennarelli TA, Malangoni MA, Ramenofsky ML (1989). Organ injury scaling: spleen, liver, and kidney. J Trauma.

[2] Croce MA, Fabian TC, Kudsk KA, Baum SL, Payne LW, Mangiante EC, Britt LG (1991). AAST organ injury scale: correlation of CT-graded liver injuries and operative findings. J Trauma.

[3] Rizoli SB, Brenneman FD, Hanna SS, Kahnamoui K (1996). Classification of liver trauma. HPB Surg.

[4] Coccolini F, Catena F, Moore EE, Ivatury R, Biffl W, Peitzman A (2016). WSES classification and guidelines for liver trauma. World J Emerg Surg..

[5] Coccolini F, Montori G, Catena F, Kluger Y, Biffl W, Moore EE (2017). Splenic trauma: WSES classification and guidelines for adult and pediatric patients. World J Emerg Surg..

[6] Coccolini F, Stahel PF, Montori G, Biffl W, Horer TM, Catena F (2017). Pelvic trauma: WSES classification and guidelines. World J Emerg Surg.

